# Building the bodily self‐awareness: Evidence for the convergence between interoceptive and exteroceptive information in a multilevel kernel density analysis study

**DOI:** 10.1002/hbm.24810

**Published:** 2019-10-14

**Authors:** Gerardo Salvato, Fabian Richter, Lucas Sedeño, Gabriella Bottini, Eraldo Paulesu

**Affiliations:** ^1^ Department of Brain and Behavioural Sciences University of Pavia Pavia Italy; ^2^ Centre of Cognitive Neuropsychology ASST Grande Ospedale Metropolitano, Niguarda Hospital Milan Italy; ^3^ NeuroMI, Milan Center for Neuroscience Milan Italy; ^4^ Department of Psychology Universität zu Köln Cologne Germany; ^5^ Laboratory of Experimental Psychology and Neuroscience (LPEN) Institute of Translational and Cognitive Neuroscience (INCyT), INECO Foundation, Favaloro University Buenos Aires Argentina; ^6^ National Scientific and Technical Research Council (CONICET) Buenos Aires Argentina; ^7^ Department of Psychology University of Milano‐Bicocca Milan Italy; ^8^ IRCCS Istituto Ortopedico Galeazzi Milan Italy

**Keywords:** bodily self, body awareness, body ownership, interoception

## Abstract

Exteroceptive and interoceptive signals shape and sustain the bodily self‐awareness. The existence of a set of brain areas, supporting the integration of information coming from the inside and the outside of the body in building the sense of bodily self‐awareness has been postulated, yet the evidence remains limited, a matter of discussion never assessed quantitatively. With the aim of unrevealing where in the brain interoceptive and exteroceptive signals may converge, we performed a meta‐analysis on imaging studies of the sense of body ownership, modulated by *external* visuotactile stimulation, and studies on interoception, which involves the self‐awareness for *internal* bodily sensations. Using a multilevel kernel density analysis, we found that processing of stimuli of the two domains converges primarily in the supramarginal gyrus bilaterally. Furthermore, we found a right‐lateralized set of areas, including the precentral and postcentral, and superior temporal gyri. We discuss these results and propose this set of areas as ideal candidates to match multiple body‐related signals contributing to the creation of a multidimensional representation of the bodily self.

## 1 INTRODUCTION

Bodily self‐awareness has been defined as the feeling that conscious experiences are bound to the self and are experiences of a unitary entity (Berlucchi & Aglioti, 2010; Blanke, 2012; Blanke, Slater, & Serino, 2015; Legrand, 2006, 2010). It is a multidimensional construct, involving several aspects such as the experience of owning a body, the perception of visceral signals coming from our own body, as well as feeling the body in space, or the agency over our actions (Berlucchi & Aglioti, 2010; Blanke, 2012; Blanke et al., 2015). Both exteroceptive and interoceptive information have been considered essential drivers for the sense of self, and most such research has focused on the investigation of one of these two domains in an exclusive manner (Park & Blanke, 2019).

The pivotal role of the exteroceptive information for the bodily self‐awareness has been mainly demonstrated throughout visuotactile experimental paradigms. One such example comes from the famous rubber hand illusion (RHI) paradigm, the visuotactile multisensory conflict generated when a synchronous brushing is administered to someone's hand, occluded from vision, and on a visible nearby rubber hand: under such circumstances, subjects “feel touches on the rubber hand” as if this was belonging their body (Botvinick & Cohen, 1998). This visuotactile mismatch is also effective between the participant's face and another person's face (enfacement illusion; Sforza, Bufalari, Haggard, & Aglioti, 2010), and a virtual body and the own body (full body illusion; Lenggenhager, Tadi, Metzinger, & Blanke, 2007).

On the other hand, interoceptive signals also play a role in the construction of a coherent sense of the self (Craig, 2002; Critchley & Harrison, 2013). Interoception has been mainly studied via paradigms like the heartbeat perception, but there are several other sources of information for different bodily sensations, which have been investigating using self‐evaluated assessments (interoceptive sensibility) and behavioral tests (interoceptive accuracy; Garfinkel, Seth, Barrett, Suzuki, & Critchley, 2015).

Signals coming from the inside and outside of the body may interact, and the interplay between the two domains is currently unclear. From a behavioral point of view, Tsakiris, Jimenez, and Costantini (2011) have shown that interoceptive awareness may modulate the malleability of the sense of the self. They administered the RHI paradigm to a group of subjects showing low or high interoceptive accuracy, as measured by the Heartbeat Perception task, to find the counterintuitive finding that the less interoceptively accurate participants had a stronger incorporation effect of the rubber hand. Later, Suzuki, Garfinkel, Critchley, and Seth (2013) have shown that both subjective and objective measures of virtual‐hand ownership are enhanced by cardio‐visual feedback in time with the actual heartbeat, as compared to asynchronous feedback. They also have shown that these measures, correlated with individual differences in interoceptive sensitivity, were modulated by the integration of proprioceptive signals instantiated using real‐time visual remapping of finger movements of the virtual hand (Suzuki et al., 2013).

Although it is not clear whether a supramodal form of bodily self‐awareness exists, this evidence definitively suggests the existence of forms of integration between interoceptive and exteroceptive signals. While it is possible that integration of these domains might occur by forms of structural and functional connectivity of separate specialized regions, the logic of the human brain organization also allows one to hypothesize the existence of higher‐order neural systems where signals of the different sources are integrated (Zeki & Shipp, 1988).

To date, a quantitative investigation on this hypothesis and differences and commonalities between the two domains is lacking. Moseley, Gallace, and Spence (2012) have proposed a theoretical framework for the interaction between interoceptive and exteroceptive information in the bodily self‐awareness. They proposed the existence of a *body matrix*, a set of neural structures that “serve to maintain the integrity of the body at both the homeostatic and psychological levels and to adapt to changes in our body structure and orientation” (Moseley et al., 2012). They have also hypothesized that the posterior parietal and insular cortices may play an important role in the body matrix. In a more recent theoretical review (Park & Blanke, 2019), it has been suggested the existence of at least two subnetworks supporting the integration between interoceptive and exteroceptive signals in bodily self‐awareness: a premotor cortex—intraparietal sulcus—insula network preferentially processing signals for self‐identification, and a temporal parietal junction—posterior cingulate cortex—intraparietal sulcus network preferentially processing signals for self‐location. Interestingly, the authors have proposed an overlap of these two subnetworks in the intraparietal sulcus (Park & Blanke, 2019). However, explicit evidence on the anatomical localization of cortical regions linking exteroceptive and interoceptive signals is limited and discrepant to what previously hypothesized. Recently, Blefari et al. (2017) in an fMRI experiment administered the full body illusion paradigm coupled with cardiovascular response and heartbeat awareness task to a group of healthy volunteers: they found and proposed that the Rolandic operculi, in coordinates falling inside ventral premotor cortex, may be the critical regions subserving the integration of external and internal bodily signals. This interesting finding, in line with early fMRI reports on the RHI paradigm (Ehrsson, Spence, & Passingham, 2004), is clearly in need of replication as it would move the emphasis on a premotor view of bodily self‐awareness.

### 1.1 Aim of the study

In the current study, we aimed at unveiling the neural networks underpinning exteroceptive and interoceptive signals, seeking for modality‐specific and shared systems. As said, the overarching aim of this work was to test the existence of a shared neural system subtending the making of bodily self‐awareness. To this scope, we submitted to a quantitative meta‐analysis, the available neuroimaging studies concerning body ownership (external visuotactile information) and interoception (internal physiological information), which included psychological illusions involving diverse body parts and different approaches to assess interoception. We first performed two separate meta‐analyses investigating which brain regions are associated with the sense of body ownership and the interoceptive awareness according to these paradigms. We then examined whether body ownership and interoception share common neural substrates. Based on previous findings, we hypothesized to find evidence for a role of high‐order associative areas, for example, the parietal regions, where the merging of external and internal information is made possible by the convergence of different inputs. Because of the specific choice made on the to‐be meta‐analyzed materials, we will argue that any such regions may contribute to the building of a bodily self‐awareness.

## 2 METHODS

### 2.1 Study selection

To select relevant neuroimaging studies concerned with brain activity related to interoception or body awareness, an extensive database search of peer‐reviewed functional neuroimaging studies with no initial restrictions (regarding the type of publication or publication date) was conducted. We relied on the following sources: MEDLINE library, life science journals, and online books indexed in PubMed. Also, we reviewed the reference lists of these articles to find additional reports and used the Sleuth Software (BrainMap Development Team, Version 2.0.3, Research Imaging Center, University of Texas Health Science Center at San Antonio) to search the BrainMap database for published functional neuroimaging experiments.

The data sets and associated contrasts included in this meta‐analysis, met the following inclusion criteria: (a) description of standard Talairach and Tournoux (1988), or MNI coordinates (to enable comparison of reported peak activation across studies); (b) samples composed of unmedicated and untrained healthy adults; (c) corrected thresholds of significance established at a whole‐brain level; (d) measurement of regional cerebral blood flow (e.g., through PET) or blood oxygenation (e.g., through fMRI); and (e) tasks tapping core processes of interoception or body ownership without assessing related high‐level processes or pursuing more specific goals (such as correlations and/or interactions with other psychological constructs or demographic features).

Unlike a recent meta‐analysis on interoception (Schulz, 2016), the present one was not limited to cardioception and included studies with a variety of tasks. We adopted this inclusive criterion because our study's aim was to compare the awareness of bodily signals (without limiting these to cardiac activity) and the ability to self‐recognize bodily information. Likewise, the meta‐analysis on body ownership included a variety of tasks manipulating the sense of body ownership.

For body ownership, the key words used to search the databases were as follows: “fMRI” <OR>“functional neuroimaging” <OR>“PET” <OR>“neural basis” <OR>neural correlate <AND>“body ownership” <OR>“body disownership” <OR> “rubber hand illusion” <OR>“virtual hand illusion.”

Following this search, our data set included 286 participants drawn from 39 contrasts derived from 16 studies.

The key words for interoception were as follows: “fMRI” <OR>“functional neuroimaging” <OR>“PET” <OR>“neural basis” <OR>neural correlate <AND>“interoception” <OR>“cardioception” <OR>“visceral perception” <OR>“body emotions” <OR>“bodily emotions” <OR>“heartbeat” <OR>“heartbeat detection.” These criteria resulted in a total of 770 participants and 73 contrasts across 40 studies (see Table 1 for lists of included studies).

**Table 1:** List of studies included on Body Ownership and Interoception included in the meta‐analysis

First author (year)	Title	Modality	Participants number	Total contrasts	Contrasts used
**Body ownership**					
Bekrater‐Bodmann et al. (2014)	The importance of synchrony and temporal order of visual and tactile input for illusory limb ownership experiences—An fMRI study applying virtual reality	fMRI	25	9	1
Brozzoli, Gentile, and Ehrsson (2012)	That's near my hand! Parietal and premotor coding of hand‐centered space contributes to localization and self‐attribution of the hand	fMRI	16	10	3
Ehrsson et al. (2004)	That's my hand! Activity in premotor cortex reflects feeling of ownership of a limb	fMRI	18	15	4
Ehrsson, Holmes, and Passingham (2005)	Touching a rubber hand: Feeling of body ownership is associated with activity in multisensory brain areas	fMRI	15	6	1
Gentile, Guterstam, Brozzoli, and Ehrsson (2013)	Disintegration of multisensory signals from the real hand reduces default limb self‐attribution: An fMRI study	fMRI	15	37	3
Gentile, Björnsdotter, Petkova, Abdulkarim, and Ehrsson (2015)	Patterns of neural activity in the human ventral premotor cortex reflect a whole‐body multisensory percept	fMRI	16	4	1
Guterstam, Gentile, and Ehrsson (2013)	The invisible hand illusion: multisensory integration leads to the embodiment of a discrete volume of empty space	fMRI	14	18	1
Guterstam, Björnsdotter, Gentile, and Ehrsson (2015)	Posterior cingulate cortex integrates the senses of self‐location and body ownership	fMRI	15	4	1
Ionta et al. (2011)	Multisensory mechanisms in temporo‐parietal cortex support self‐location and first‐person perspective	fMRI	22	1	1
Limanowski and Blankenburg (2016)	That's not quite me: limb ownership encoding in the brain	fMRI	13	33	3
Limanowski and Blankenburg (2015)	Network activity underlying the illusory self‐attribution of a dummy arm	fMRI	20	24	6
Limanowski and Blankenburg (2018)	Fronto‐parietal brain responses to visuotactile congruence in an anatomical reference frame.	fMRI	20	13	1
Petkova et al. (2011)	From part‐to whole‐body ownership in the multisensory brain	fMRI			
	Exp 1		26	13	2
	Exp 2		20	29	2
	Exp 3		20	48	1
Preston and Ehrsson (2016)	Illusory obesity triggers body dissatisfaction responses in the insula and anterior cingulate cortex	fMRI	32	17	2
Tsakiris, Hesse, Boy, Haggard, and Fink (2007)	Neural signatures of body ownership: A sensory network for bodily self‐consciousness	PET	10	10	4
Tsakiris, Longo, and Haggard (2010)	Having a body versus moving your body: Neural signatures of agency and body‐ownership	fMRI	19	18	2
**Interoception**					
Araujo, Kaplan, Damasio, and Damasio (2015)	Neural correlates of different self‐domains	fMRI	19	6	1
Avery et al. (2015)	A common gustatory and interoceptive representation in the human mid‐insula	fMRI	20	3	1
Bauer, Díaz, Concha, and Barrios (2014)	Sustained attention to spontaneous thumb sensations activates brain somatosensory and other proprioceptive areas	fMRI	34	6	1
Becker, Schmälzle, Flaisch, Renner, and Schupp (2014)	Thirst and the state‐dependent representation of incentive stimulus value in human motive circuitry	fMRI	24	2	1
Binks, Evans, Reed, Moosavi, and Banzett (2014)	The time‐course of cortico‐limbic neural responses to air hunger	fMRI	8	2	1
Blefari et al. (2017)	Bilateral Rolandic operculum processing underlying heartbeat awareness reflects changes in bodily self‐consciousness	fMRI	16	5	1
Brannan et al. (2001)	Neuroimaging of cerebral activations and deactivations associated with hypercapnia and hunger for air	fMRI	9	3	3
Cameron and Minoshima (2002)	Regional brain activation due to pharmacologically induced adrenergic interoceptive stimulation in humans	PET	24	1	1
Caseras et al. (2013)	Anatomical and functional overlap within the insula and anterior cingulate cortex during Interoception and phobic symptom provocation	fMRI	46	2	1
Coen et al. (2009)	Negative mood affects brain processing of visceral sensation	fMRI	12	4	1
Critchley (2004)	Neural systems supporting interoceptive awareness	fMRI	17	2	1
Denton et al. (1999a)	Correlation of regional cerebral blood flow and change of plasma sodium concentration during genesis and satiation of thirst	PET	10	1	1
Denton et al. (1999b)	Neuroimaging of genesis and satiation of thirst and an interoceptor‐driven theory of origins of primary consciousness	PET	10	4	4
Egan et al. (2003)	Neural correlates of the emergence of consciousness of thirst	fMRI and PET	10	5	5
Ernst, Northoff, Böker, Seifritz, and Grimm (2013)	Interoceptive awareness enhances neural activity during empathy	fMRI	18	3	1
Evans et al., 2002	BOLD fMRI identifies limbic, paralimbic, and cerebellar activation during air hunger	fMRI	6	1	1
Farb, Segal, and Anderson (2013)	Mindfulness meditation training alters cortical representations of interoceptive attention	fMRI	16	3	1
Farrell et al. (2006)	Unique, common, and interacting cortical correlates of thirst and pain	PET	10	3	3
Haase et al. (2015)	A pilot study investigating changes in neural processing after mindfulness training in elite athletes	fMRI	7	3	3
Haase et al. (2016)	When the brain does not adequately feel the body: Links between low resilience and interoception	fMRI	46	4	1
Hassanpour et al. (2018)	The insular cortex dynamically maps changes in cardiorespiratory interoception	fMRI	23	2	1
Immordino‐Yang, Yang, and Damasio (2014)	Correlations between social–emotional feelings and anterior insula activity are independent from visceral states but influenced by culture	fMRI	15	3	3
Isaev, Murphy, Guz, and Adams (2002)	Areas of the brain concerned with ventilatory load compensation in awake men	PET	10	1	1
Kuehn, Mueller, Lohmann, and Schuetz‐Bosbach (2016)	Interoceptive awareness changes the posterior insula functional connectivity profile	fMRI	12	7	2
Liotti et al. (2001)	Brain responses associated with consciousness of breathlessness (air hunger)	PET	9	2	2
May, Stewart, Migliorini, Tapert, and Paulus (2013)	Methamphetamine dependent individuals show attenuated brain response to pleasant interoceptive stimuli	fMRI	17	2	1
May, Stewart, Tapert, and Paulus (2014)	The effect of age on neural processing of pleasant soft touch stimuli	fMRI	58	2	1
Oberndorfer et al. (2013)	Greater anterior insula activation during anticipation of food images in women recovered from anorexia nervosa versus controls	fMRI	12	4	1
Perini, Morrison, and Olausson (2015)	Seeking pleasant touch: Neural correlates of behavioral preferences for skin stroking	fMRI	18	8	4
Pollatos, Schandry, Auer, and Kaufmann (2007)	Brain structures mediating cardiovascular arousal and interoception	fMRI	20	4	3
Simmons et al. (2013)	Keeping the body in mind: Insula functional organization and functional connectivity integrate interoceptive. Exteroceptive, and emotional awareness	fMRI	14	3	1
Stern et al. (2017)	Neural correlates of Interoception: Effects of interoceptive focus and relationship to dimensional measures of body awareness	fMRI	19	7	3
Stewart, Parnass, May, Davenport, and Paulus (2013)	Altered frontocingulate activation during aversive interoceptive processing in young adults transitioning to problem stimulant use	fMRI	29	6	1
Stewart et al. (2014)	You are the danger: Attenuated insula response in methamphetamineusers during aversive interoceptive decision‐making	fMRI	22	3	1
Stewart, Juavinett, May, Davenport, and Paulus (2015)	Do you feel alright? Attenuated neural processing of aversive interoceptive stimuli in current stimulant users	fMRI	15	4	2
Strigo et al. (2013)	Altered insula activation during pain anticipation in individuals recovered from anorexia nervosa: Evidence of interoceptive Dysregulation	fMRI	22	3	2
Terasawa, Fukushima, and Umeda (2013)	How does interoceptive awareness interact with the subjective experience of emotion	fMRI	18	6	4
Tracy et al. (2007)	Functional magnetic resonance imaging analysis of attention to one's heartbeat	fMRI	17	5	5
Wang et al. (2008)	Gastric distention activates satiety circuitry in the human brain	fMRI	18	1	1
Zaki, Davis, and Ochsner (2012)	Overlapping activity in anterior insula during	fMRI	16	4	1

### 2.2 Multilevel kernel density analysis

For this study, we used the multilevel kernel density analysis (MKDA; Kober et al., 2008; Kober & Wager, 2010; Wager, Lindquist, & Kaplan, 2007; Wager, Lindquist, Nichols, Kober, & Van Snellenberg, 2009), which allows one to detect robust effects and to establish the level of consistency across findings (Costafreda, 2009; Lindquist, Satpute, Wager, Weber, & Barrett, 2016; Schurz, Radua, Aichhorn, Richlan, & Perner, 2014). The technique reduces variability between studies in search of convergent findings across a set of studies, to average results to identify brain areas that show consistent activity (Liang, Zou, He, & Yang, 2013; van den Heuvel & Sporns, 2013). The MKDA emphasizes the multilevel hierarchy of the meta‐analytic input data by treating activation peaks as nested within a given contrast. The resulting contrast maps—instead of individual peaks—are the unit of analysis and through this, two levels of analysis (within‐contrasts and between contrasts) are created. Therefore, the MKDA summarizes consistency across studies rather than across peak coordinates. An important consequence of this approach is that any single study that reports a large number of nearby peaks (due to differences in style of how regional effects are reported, voxel size, thresholding, or low spatial smoothness in the data) cannot have an excessive weight on the final results (Wager et al., 2009; for further explanations see also Supporting Information).

More specifically, the MKDA performs multiple nested analyses on individual peaks by: (a) nesting peak activation coordinates within contrasts, and contrasts within studies; (b) modeling the variability across peaks within a contrast, rather than just counting all the peaks without taking into account from which contrast or study the peaks come from—so that true effect sizes are assumed to vary between studies; (c) assessing statistical significance density maps by comparing against the null hypothesis whereby activated regions are randomly distributed throughout the whole brain (Kober & Wager, 2010; Wager et al., 2009).

To this end, one contrast indicator map (CIM) is created for each contrast, by convolving a 10‐mm spherical kernel around each peak reported in this contrast. The CIMs are averaged and weighted by the square root of the sample size of each contrast so that studies with fewer participants are given less weight, while reports with a larger number of participants are given more weight. Additionally, CIMs are weighted with 0.75 for fixed‐effects versus 1.00 for random effects analysis to reduce the impact of fixed‐effect studies (Wager et al., 2007, 2009; see also Supporting Information).

The resulting Summary Density Map built up of the individual CIMs reflects the proportion of contrasts yielding activations near each voxel. Hence, the crucial measure of interest is the number of contrasts that produced activation near a voxel, rather than the number of individual activation peaks.

**Figure 1:**
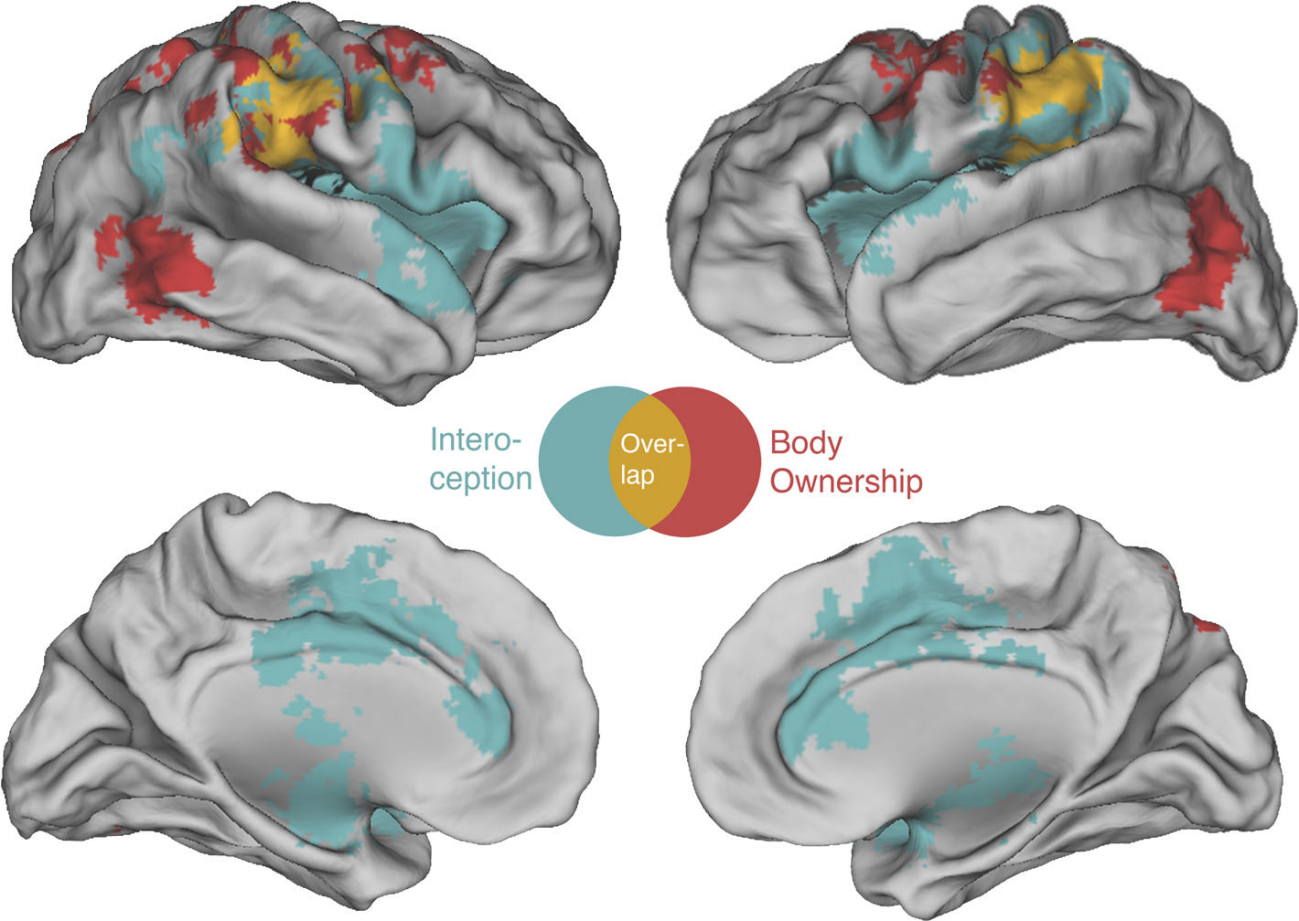
Results of the conjunction analysis. The upper panel shows the body ownership (red) and interoception (light blue) areas of activations. The voxels of overlap between the two functions are shown in yellow. Voxels are family‐wise error rate (FWER) corrected at *p* < .05 with primary alpha levels of .05 for spatial extent. The lower figure quantifies overlapping areas following the AAL atlas. *Z*‐scores indicate the maximal weighted percentage of contrasts in an area, separately for both meta‐analyses. Only conjoint activity resulting in a cluster size with a minimum of 10 voxels is reported. Voxel‐size is 2 x 2 x 2 mm; SMG, supramarginal gyrus; R, right; L, left [Color figure can be viewed at http://wileyonlinelibrary.com]

The general null hypothesis states that peak coordinates of activated regions are randomly distributed across the gray matter of the standard brain. To identify voxels with activations that exceed the frequency expected by chance, a threshold derived from a Monte Carlo Simulation with 5,000 iterations per analysis was used. Building on this, MKDA identified maps of activated clusters according to a “height” or “extent” based threshold. The height‐based threshold encloses voxels that have proportions of contrasts inside the 10 mm kernel regions that exceed the maximum expected over the entire brain by chance (*p* < .05, family wise error rate—FWER corrected). To determine a cluster extent‐based threshold, the largest cluster of contiguous voxels was saved after each Monte Carlo iteration (with primary alpha levels of .001, .01, and .05) and secondary FWER‐corrected for spatial extent at *p* < .05. Beyond that, a combined map of voxels meeting both criteria (height [*p* < .05] and extent [primary alpha level: *p* < .05]) was computed using SPM8 contiguity assessment procedures (see Figure 1; Kober et al., 2008; Schulz, 2016). In conclusion, the statistics reported in text and tables as the “*z*‐value” (Kober et al., 2008) correspond to the proportion of contrasts that activated within 10 mm (kernel region) of that voxel, weighted by the sample size and study design analysis (fixed or random effects; Buhle et al., 2014; Denny, Kober, Wager, & Ochsner, 2012; Kober et al., 2008; Mende‐Siedlecki, Said, & Todorov, 2013). Thus, *z*‐values (maximum statistic values) are based on weighted CIMs rather than studies or peaks and represent the weighted percentage of CIMs that reported activation in each cluster (Kober et al., 2008; see also Supporting Information). The results were visualized using Caret Software (version 5.65, Van Essen Laboratory, Saint Louis; http://brainvis.wustl.edu). Activation maps for both domains containing the combined results from extent‐ and height‐based thresholds (*p* < .05, FWER‐corrected) were projected onto the left and right hemispheres of the PALS atlas (Population‐Average, Landmark and Surface‐based atlas, Van Essen Laboratory, Saint Louis; http://sumsdb.wustl.edu/sums/humanpalsmore.do) using MNI coordinate space. Axial cut planes of the overlap areas on a template brain image were created using the Mango software (version 4.0.1, Research Imaging Institute, UTHSCSA).

### 2.3 Statistical analyses to test specific effects

To extract the areas activated by both domains (interoception and body ownership), a conjunction analysis was conducted on the two combined (height‐ and extend‐based) MKDA maps (at 0.05 FWER‐corrected), as described by Nichols, Brett, Andersson, Wager, and Poline (2005) using MATLAB (The MathWorks, Inc., Natick, MA), and SPM8 (Statistical Parametric Maps, Wellcome Department of Cognitive Neurology, http://www.fil.ion.ucl.ac.uk/spm). Only conjoint activity resulting in a cluster‐size with a minimum of 10 voxels is reported. The clusters were analyzed using the xjView toolbox (http://www.alivelearn.net/xjview/).

Furthermore, we compared the activation of body ownership and interoception by subtraction analysis in MKDA: separate maps constructed for both domains were subtracted to yield difference maps. The same procedure was employed during the Monte Carlo randomization to establish a threshold for significant differences.

## 3 RESULTS

### 3.1 Body ownership

For studies employing tasks addressing the concept of body ownership, peak areas of high concordance were detected in the inferior temporal gyri spanning to the inferior occipital lobes (right, *z* = 0.2; left, *z* = 0.18). Further, we found bilateral clusters in the inferior parietal lobes also involving the postcentral gyri with cluster centers in the supramarginal gyri (right, *z* = 0.14; left, *z* = 0.17). Additional cluster centers were located in the precentral gyri (right, *z* = 0.15; left, *z* = 0.15), the cerebellum (right cerebellar tonsil, *z* = 0.16; right declive, *z* = 0.09; left declive = 0.08), the right parietal inferior lobe (*z* = 0.15), the right superior parietal lobe (*z* = 0.12), and the insular cortices (right, *z* = 0.08; left, *z* = 0.06; see Table 2 and Figure 1).

**Table 2:** Results of the meta‐analysis on body ownership

Area	*x*	*y*	*z*	Brodmann area	Voxels	Volume (mm^3^)	Maxstat (*z*)
*Height threshold (p < .05, family wise error rate—FWE corrected)*							
Right precentral gyrus	48	4	40	6	1	8	0.15
Left inferior parietal lobe	−60	−30	28	40	1	8	0.17
Right inferior temporal gyrus	50	−56	−6	19	28	224	0.20
Left inferior temporal gyrus	−46	−70	−2	37	21	168	0.18
Left fusiform gyrus	−40	−70	−10	19	46	368	0.18
Right cerebellum (cerebellar tonsil)	28	−72	−36		1	8	0.16
*Extent threshold (primary alpha level of p < .001)*							
Left precentral gyrus	−50	0	34	6	236	1,888	0.15
Right inferior parietal lobe	54	−22	26	40	210	1,680	0.14
Right inferior parietal lobe	54	−28	46	40	399	3,192	0.15
*Extent threshold (primary alpha level of p < .01)*							
Left cerebellum (Declive)	−32	−70	−16		13	104	0.08
*Extent threshold (primary alpha level of p < .05)*							
Right insula	42	−14	20	13	25	200	0.07
Left insula	−42	−24	20	13	61	488	0.06
Left inferior parietal lobe	−44	−28	38	40	43	344	0.05
Right insula	42	−32	20	13	77	616	0.05
Left inferior parietal lobe	−58	−34	44	40	49	392	0.05
Right insula	58	−36	24	13	213	1,704	0.08
Right superior parietal lobe	34	−52	52	7	1,366	10,928	0.12
Right cerebellum (Declive)	34	−62	−24		2,275	18,200	0.09

*Note*: Stereotactic coordinates for the most consistent clusters across all body ownership studies according to a “height” and “extent” based thresholds. Height‐based threshold encloses voxels that have proportions of contrasts inside the 10 mm kernel regions that exceed the maximum expected over the entire brain by chance (*p* < .05, family wise error Rate—FWER corrected). Extent‐based threshold encloses contiguous voxels outside the 10 mm of the clusters for the height‐based threshold that showed greater activation than would be expected at a given level of chance (*p* < .001), and which are secondary FWER‐corrected for spatial extent at *p* < .05.

### 3.2 Interoception

The meta‐analysis of interoception showed the most significant cluster in the right insula (*z* = 0.26) followed by the left insula (*z* = 0.22), the left inferior parietal gyrus (*z* = 0.17), the left medial frontal gyrus (*z* = 0.17), the right thalamus (pulvinar, *z* = 0.15; medial dorsal nucleus, *z* = 0.09), the right postcentral (*z* = 0.14), and precentral gyrus (*z* = 0.11), the cingulate gyrus (midcingulate, *z* = 0.14; anterior cingulate, *z* = 0.13), the right midbrain (*z* = 0.1), and the right medial temporal gyrus (*z* = 0.06; see Table 3 and Figure 1).

**Table 3:** Results of the meta‐analysis on interoception

Area	*x*	*y*	*z*	Brodmann area	Voxels	Volume (mm^3^)	Maxstat (*z*)
*Height threshold (p < .05, family wise error rate—FWE corrected)*							
Right insula	40	8	4	13	755	6,040	0.26
	40	10	4		559		0.26
	40	−6	8		150		0.19
	36	6	12		46		0.19
Mid cingulate gyrus	2	8	42	24	1	8	0.14
Mid cingulate gyrus	−4	6	40	24	1	8	0.14
Mid cingulate gyrus	0	6	38	24	1	8	0.14
Left insula	−40	4	4	13	269	2,152	0.22
Left medial frontal gyrus	2	0	48	6	132	1,056	0.17
	−2	−2	44		32		0.17
	4	2	48		100		0.16
Mid cingulate gyrus	−4	−10	40	24	1	8	0.14
Right thalamus (Pulvinar)	18	−24	8		14	112	0.15
Right postcentral gyrus	52	−24	34	2	1	8	0.14
Left inferior parietal lobe	−56	−26	24	40	2	16	0.14
Left inferior parietal lobe	−58	−34	30	40	36	288	0.17
Left inferior parietal lobe	−52	−34	26	40	3	24	0.14
*Extent threshold (primary alpha level of p < .001)*							
Anterior cingulate	0	30	16	24	163	1,304	0.13
Right insula	44	−2	10	13	1,823	14,584	0.13
Right precentral gyrus	22	−30	62	4	191	1,528	0.11
*Extent threshold (primary alpha level of p < .01)*							
Right midbrain (substantia nigra)	14	−20	−8		423	3,384	0.10
*Extent threshold (primary alpha level of p < .05)*							
Thalamus (medial dorsal Ncl.)	2	−10	16		9,618	76,944	0.09
Right mid temporal gyrus	52	−62	20	19	28	224	0.06

*Note*: Stereotactic coordinates for the most consistent clusters across all interoception studies according to a “height” and “extent” based thresholds. Height‐based threshold encloses voxels that have proportions of contrasts inside the 10 mm kernel regions that exceed the maximum expected over the entire brain by chance (*p* < .05, family wise error Rate—FWER corrected). Extent‐based threshold encloses contiguous voxels outside the 10 mm of the clusters for the height‐based threshold, which showed greater activation than would be expected at a given level of chance (*p* < .001), and which are secondary FWER‐corrected for spatial extent at *p* < .05.).

### 3.3 Contrast analyses between body ownership and interoception

The analysis of the contrast map Body Ownership > Interoception revealed that the left inferior occipital gyrus (*z* = 0.17) and the right middle occipital gyrus (*z* = 0.17) were associated with body ownership (see Supporting Information). These regions were in stereotactic locations consistent with the extrastriate visual areas involved in the visual processing of human bodies.

Furthermore, the contrast map Interoception > Body Ownership revealed that several deep cortical and subcortical regions, such as the left and right insula (*z* = 0.19; *z* = 0.23), the right (*z* = 0.13), and left (*z* = 0.1) thalamus, the left globus pallidus (*z* = 0.13), and the mid‐cingulate gyrus (*z* = 0.15) as well as the anterior cingulate gyrus (*z* = 0.12), were more likely associated with interoception (see Supporting Information).

### 3.4 Areas shared by the sense of body ownership and interoception

Figure 1 illustrates the MKDA maps (conjoint of height and extent threshold) of body ownership and interoception tasks on a single template (PALS atlas; Van Essen, 2005): the largest areas of overlap were seen bilaterally in the inferior parietal lobules, in particular in the supramarginal gyri (SMG) spanning to the postcentral gyri. Further areas significantly activated by both meta‐analyses are the rolandic opercula, the right precentral gyrus, the superior temporal lobule, and the inferior parietal lobe.

**Figure 2:**
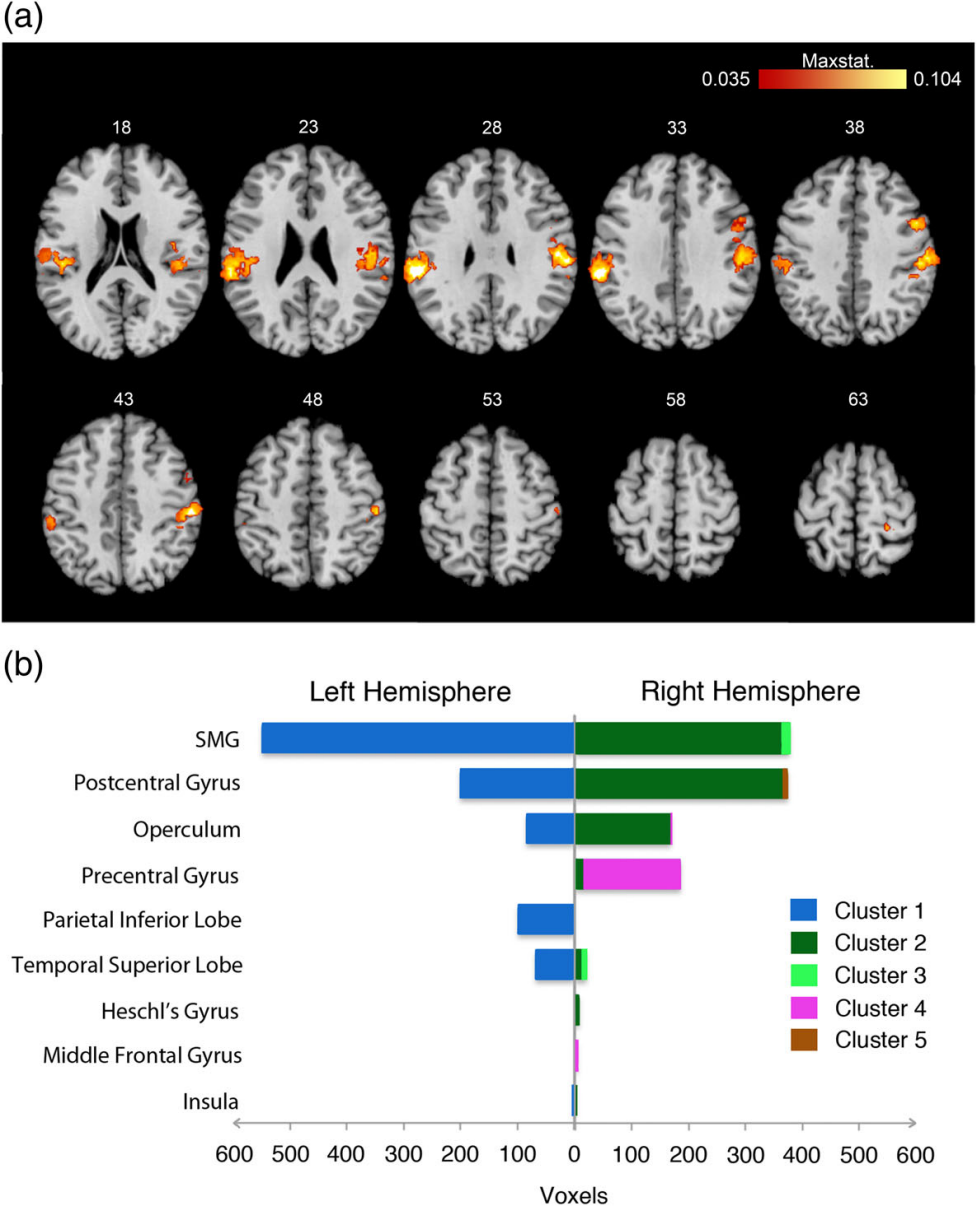
Results of the cluster analysis on the overlap. Panel A shows axial brain slices of the overlapping areas mapped on a standard brain template. Numbers above the slices indicate *z*‐coordinates. Maxstat is the mean *z*‐value of both meta‐analyses. Panel B shows the brain areas (following AAL atlas) covered by the respective clusters. Voxel‐size is 2 x 2 x 2 mm; SMG, supramarginal gyrus [Color figure can be viewed at http://wileyonlinelibrary.com]

A cluster‐based analysis of such convergence revealed by the conjunction analysis (*p* < .05, FWER‐corrected, Figure 2) unveiled two large clusters with their cluster centers in the right and left SMG, respectively (left SMG: 1,077 voxels; *z* = 0.14; right SMG: 1,003 voxels; *z* = 0.12).^1^ Additionally, three smaller clusters were found in the right hemisphere. The maximum activation of the respective cluster was hereby found in the precentral gyrus (182 voxels; *z* = 0.11), the superior temporal gyrus (28 voxels; *z* = 0.07), and the postcentral gyrus (12 voxels; *z* = 0.08; see Table 4).

**Table 4:** Results of the cluster analysis

Area	*x*	*y*	*z*	Brodmann area	Cluster size (voxels)	Maxstat (*z*)
Left supramarginal gyrus (Cluster 1)	−60	−30	28	40	1,077	0.14
Right supramarginal gyrus (Cluster 2)	56	−24	42	2	1,003	0.12
Right superior temporal gyrus (Cluster 3)	62	−40	22	13	28	0.07
Right precentral gyrus (Cluster 4)	48	4	40	6	182	0.11
Right postcentral gyrus (Cluster 5)	24	−38	62	5	12	0.08

*Note*: Stereotactic coordinates for the most consistent clusters of convergence between interoception and body ownership activation studies (*p* < .05, family wise error rate—FWER corrected.

## 4 DISCUSSION

We continuously receive different signals from our body, coming from both the inside and outside multisensory channels. Are there specific cerebral areas permitting the convergence of the two domains? Evidence on the topic was scanty with suggestions, based on qualitative reviews of the available evidence, that these levels of multisensory integration might be supported by the insular, posterior parietal cortex (Moseley et al., 2012; Park & Blanke, 2019), while one explicit experiment on the matter pointed to ventral premotor cortex (Blefari et al., 2017).

Here, we instead quantified the available evidence on where the internal and external signals may be integrated and demonstrated the specific importance of the inferior parietal cortex of both hemispheres, comprising the parietal portion of the Rolandic operculum and insula, together with right‐lateralized areas such as the postcentral, precentral, and superior temporal gyri. In what follows, we will discuss in an orderly manner the brain regions mainly associated with body ownership, interoception, and the areas of convergence between the two domains.

### 4.1 Body ownership

Our meta‐analysis identified a compound set of occipitotemporal, premotor, parietal, and insular regions contributing to the sense of body ownership, consistent with the nature of the triggering paradigms. In keeping with previous studies and a very recent meta‐analysis, this network most likely integrates bodily signals across diverse sensory channels (Grivaz, Blanke, & Serino, 2017).

The areas with the most significant clusters were the right inferior temporal gyrus and the left inferior occipital lobe. These occipitotemporal regions have been identified as part of the body self‐recognition network. The activity of this network, and in particular of the Fusiform Body Area together with the Extrastriate Body Area, has been associated with the perception and identification of bodies and their parts. More specifically, these occipital areas would be involved in the implicit processing of morphological features peculiar of a person's body, which are further processed by more anterior regions to explicitly define who a person might be (De Bellis, Trojano, Errico, Grossi, & Conson, 2017).

Another area with high convergent activation across studies was the left SMG. This parietal region has been associated with the decoding of self‐location (Guterstam et al., 2015) and perceiving limbs in space in a body‐centered reference (Brozzoli et al., 2012). In particular, during the RHI paradigm, being the real hand position remapped onto a prosthetic hand, such remapping associated with activity in the posterior parietal cortex closely reflects changes in the position sense of the arm (Brozzoli et al., 2012). Furthermore, the SMG is a part of the neuronal network integrating visuotactile input applied to the hand (Gentile et al., 2013), and other findings indicate that this region also receives proprioceptive inputs (Berlucchi & Vallar, 2018; Freedman & Ibos, 2018; Paulesu, Frackowiak, & Bottini, 1997; Whitlock, 2017).

Our results further suggest that the right precentral gyrus plays a role in body ownership. Such an area is supposed to be implicated in the experimental manipulation of the sense of ownership concerning peripersonal space remapping (Brozzoli et al., 2012), and the multisensory integration of tactile‐proprioceptive and visual inputs (Ehrsson et al., 2005).

Lastly, our meta‐analysis also highlights the importance of cerebellum in body ownership. In the RHI paradigm, it has been shown that the activity of the cerebellum correlates with the perceived strength of the illusion (Ehrsson et al., 2004; Gentile et al., 2013; Petkova et al., 2011). Furthermore, it also contributes to distinguishing sensory signals generated by the self or by others, as in the case of the sense of touch (Blakemore, Frith, & Wolpert, 2001). In a recent fMRI study on anosognosia for hemiplegia, right cerebellar activation was the only distinctive finding between the veridical appreciations of voluntary motion of the nonparalyzed hand, and the delusional belief of having moved the paralyzed hand (Gandola et al., 2014). This suggests that the cerebellum might have a role in “closing the loop” in a body and action monitoring system. Moreover, the cerebellum is involved in the processing of synchrony and congruence among the senses (Blakemore et al., 2001; Ito, 2000; Miall, Weir, Wolpert, & Stein, 1993), integrating signals coming from different multisensory channels. Indeed, it has been suggested that the cerebellum is crucial for the detection of corresponding multisensory signals and the formation of cross‐modal predictions (Gentile et al., 2013).

Interestingly enough, unlike a recent meta‐analysis on body ownership (Grivaz et al., 2017), we did not find the insula as an area of convergent activation of body ownership paradigms, except when using a less conservative threshold (see Table 2). Of course, the insula is part of the somatosensory network (see review in Bottini et al., 1995; Paulesu et al., 1997) and of the pain matrix and it may participate to the motor awareness (Karnath, 2005). One may speculate that, because the insula is involved in somatosensory perception from ground up, the subtle body ownership paradigms (e.g., the RHI) are not sufficient to give a consistent activation of the structure. Indeed, the difference sought in the paradigm if between the integration of synchronous touches compared to asynchronous touches to the subjects' hand and visual stimuli given to the rubber hand. However, one may wonder why other meta‐analyses found body‐awareness clusters in that region; the only other reason, we can invoke is a methodological difference behind our studies. Unlike Grivaz et al. (2018), we did not consider peaks resulting from small‐volume corrections or ROIs, and we used a more conservative statistical threshold (FWER *p* < .05).

### 4.2 Interoception

The meta‐analysis of the studies on interoception confirmed the involvement of brain areas that have entered into common neuroscientific discourse when referring to brain foundations of bodily feelings (Craig, 2002). The area with the clusters of maximal significance in terms of peak height and spatial extent was in the bilateral insulae, widely considered the primary interoceptive cortical area (Craig, 2002; Critchley, 2004; Pollatos et al., 2007). It has been suggested that the right (anterior) insula mediates explicit awareness of internal bodily processes (Craig, 2002; Critchley, Wiens, Rotshtein, Öhman, & Dolan, 2004), the laterality being associated with the nature of afferents. Stimuli such as air hunger, pain, cardiac perception, and visceral perception are primarily projected to the right anterior insula via vagal pathways (Craig, 2002). Instead, stimuli like the subjective sense of fullness (Stephan et al., 2003) are said to provide input primarily to the left anterior insula (Craig, 2002; Kelly et al., 2012). This is in part consistent with a recent cardiac interoception meta‐analysis showing a right hemispheric dominance for cardioception (Schulz, 2016). Our meta‐analysis was not limited to cardioception and included studies with a variety of tasks (e.g., thirst, air‐hunger, attention to spontaneous sensations, soft touch, and gastric balloon distension), possibly explaining the bilateral distribution of the clusters.

We also found highly convergent activation in the cingulate cortex (mainly the middle and anterior portions), which is—along with the insula—involved in emotional, homeostatic/allostatic, sensorimotor, and cognitive functioning (Craig, 2002; Critchley et al., 2003; Critchley, Tang, Glaser, Butterworth, & Dolan, 2005; Devinsky, Morrell, & Vogt, 1995). Resting‐state imaging showed the anterior insula to be functionally connected with the anterior cingulate cortex and the middle cingulate cortex, while the mid/posterior insula seems to be connected with the middle cingulate cortex (Taylor, Seminowicz, & Davis, 2009). Notably, in patients suffering from irritable bowel syndrome abnormal rectal‐evoked, fMRI responses in both, the insula and cingulate cortex have been identified (Davis et al., 2008).

Furthermore, the insula and the cingulate cortex are part of the so‐called salience system, which is said to detect relevant environmental changes regardless of the sensory modality employed in the task (Downar, Crawley, Mikulis, & Davis, 2002). This evidence points in the direction that conjoint activation of the cingulate cortex and the insula contributes to salience detection (Seeley et al., 2007) through task‐set maintenance (Dosenbach et al., 2007; Dosenbach, Fair, Cohen, Schlaggar, & Petersen, 2008) and sustained focal attention (Nelson et al., 2010). Thus, anterior cingulate cortex may act as an integrative hub for interoceptive perception (Kleckner et al., 2017). As different interoceptive dimensions have different neuroanatomical correlates (García‐Cordero et al., 2016), while the insula seems to be a critical region for interoception in general, anterior cingulate cortex involvement could be dependent on the type of interoceptive processing (Couto et al., 2015). Also related to interoception, we found clusters in the thalamus, a relay of the lamina‐1‐spino‐thalamo‐cortical as well as the vagal pathway projecting physiological signals to the interoceptive network (Craig, 2002).

### 4.3 Body ownership versus interoception and vice‐versa

#### 4.3.1 Body ownership > interoception

It is worth emphasizing that the occipitotemporal areas discussed above were more frequently associated with the body‐ownership paradigms rather than with the interoception ones. This distinction survived a formal statistical assessment. Prima facie, this may merely reflect the visual nature of the body ownership illusion triggering paradigms: however, the spatial consistency of these foci with visual regions specialized for the processing of body parts makes this observation less than trivial to instead suggest a crucial contribution of these cortices to the illusory perception of ownership of the RHI or the Full Body Illusion. Crucially, TMS over these areas can modulate to what extent the body and its parts are experienced as part of one's own body (De Bellis et al., 2017; Urgesi, Calvo‐Merino, Haggard, & Aglioti, 2007).

#### 4.3.2 Interoception > body ownership

As for body ownership, we also found specific associations in the case of interoception. These localized primarily in the insulae bilaterally. Interestingly, the insular cortices also receive somatosensory stimuli for exteroceptive stimulation (Bottini et al., 1995; Burton, Videen, & Raichle, 1993). Their modulation through caloric vestibular stimulation can reverse attentional hemianaesthesia (Bottini et al., 1995). The same stimulation can revert the pathological sense of disownership observed in somatoparaphrenia (Bisiach, Jisa, Oni, & Vallak, 1991; Rode et al., 1992; Salvato et al., 2016, 2018). The receptive fields of the somatosensory insular or retro‐insular neurons are vast with responses for stimuli to either side of the body or both sides of the body (Paulesu et al., 1997). These anatomo‐physiological considerations suggest a noncomplete separation between interoceptive and exteroceptive signals for the sake of the generation of a sense of ownership instead, they suggest a gradient from regions more, but not exclusively, concerned with interoception, like the insulae, to regions more concerned with exteroceptive stimulation and body ownership (see below).

A close look to Figure 1 suggests the existence of a functional anatomical pattern, with a latero‐medial gradient for the processing of exteroceptive rather than interoceptive stimuli: medial activations were mainly linked to interoception, whereas lateral activations were shared, or dominated by exteroception. This pattern reminds a similar gradient for the default mode network (DMN) and dorsal attentional network (DAN), which are both part of the brain networks observed at rest. Interestingly, the DMN has been associated with self‐referential mental activity and internally oriented emotional processing (Buckner, Andrews‐Hanna, & Schacter, 2008; Gusnard, Akbudak, Shulman, & Raichle, 2001), whereas the DAN is active during any externally directed cognitive process (Corbetta & Shulman, 2002). This scenario fits well with our findings indicating a possible implication of distinctive resting‐state networks in interoceptive (DMN internally driven activity) and the exteroceptive (DAN externally driven activity) processing.

These networks are typically anticorrelated at rest (Gao & Lin, 2012). However, the convergence of the two networks in task‐based activation patterns suggests that their anticorrelation is not an inevitable rule; the same observation suggests that indeed the two networks, or part of them, that is, the areas of convergence, may move in the same functional direction when a sense bodily self‐awareness is generated. Admittedly, this remains a suggestion, though, as a meta‐analysis, by its nature, can only define areas of anatomical convergence but not the timing of such convergence and functional coherence.

### 4.4 Convergence between body ownership and interoception

The largest areas of shared “meta‐analytical activations” were found in a bilateral cluster centered in the SMG. The most likely homolog of the human SMG is the monkey area 7b, which, to our knowledge, has never been associated with interoception. However, the same area has connections with the granular insula and more generally with the limbic system (Friedman, Murray, O'Neill, & Mishkin, 1986; Hyvärinen, 1982; Mesulam, Van Hoesen, Pandya, & Geschwind, 1977), the main region that we associated with interoception. Accordingly, it becomes less surprising our identification of the SMG as the cortical region for both body ownership and interoception. In humans, it has been demonstrated that the SMG is activated by tasks in which both ownership and interoception are required. Kashkouli Nejad et al. (2015) asked participants to direct their awareness to certain parts of their body during the fMRI scan. They found bilateral activations in SMG, which was modulated by the participants' level of experience concerning the meditative practice involving the body (Kashkouli Nejad et al., 2015). Furthermore, Heydrich et al. (2018) measured brain activity during the “cardio‐visual full body illusion” which combines interoceptive and exteroceptive signals, by providing participants with visual exteroceptive information about their heartbeat in the form of a periodically illuminated silhouette outlining a video image of the participant's body and flashing in synchrony with their heartbeat (Heydrich et al., 2018). They found that a late somatosensory evoked potential component (P45) reflected the illusory self‐identification with a virtual body, demonstrating that the combination of interoceptive and exteroceptive signals modulate activity in the parietal somatosensory cortex (Heydrich et al., 2018).

The parietal cortex also seems to play a role in integrating physiological signals, such as body temperature. Recent studies have suggested the existence of a link between the sense of body ownership and body temperature (Kammers, Rose, & Haggard, 2011; Moseley et al., 2008; Salvato, Gandola, et al., 2018; Sedda, Tonin, Salvato, Gandola, & Bottini, 2016; Tieri, Gioia, Scandola, Pavone, & Aglioti, 2017). In healthy subjects, the transient, illusory incorporation of the fake hand in RHI paradigm has been associated with a decrease of the real hand temperature (Moseley et al., 2008). Furthermore, limb temperature modifications in healthy subjects may affect the strength of the illusion of ownership toward the rubber hand (Kammers et al., 2011).

We also found a set of right‐lateralized clusters centered in the precentral, postcentral, and superior temporal gyri. Interestingly, the precentral gyrus cluster coordinates overlap with the right ventral Premotor cortex (PMv; Mayka, Corcos, Leurgans, & Vaillancourt, 2006), which supports the perception of the self in space (Grivaz et al., 2017; Serino et al., 2013). This area is also essential for sensorimotor integration (Cooke, 2004; Iacoboni, Woods, Lenzi, & Mazziotta, 1997). In the same fashion, the right superior temporal gyrus is a hot‐zone for body representation as well. Indeed, our cluster center is very adjacent to the right temporoparietal junction (rTPJ; from http://www.neurosynth.org Yarkoni, Poldrack, Nichols, Van Essen, & Wager, 2011; uniformity test and posterior probability = 0.71). It is largely recognized that the rTPJ integrates different multisensory bodily signals (visual, tactile, spatial, vestibular; Bottini et al., 2001; Ionta et al., 2011; Lopez, Halje, & Blanke, 2008). Further, the interference with the rTPJ activity through TMS made the distinction between what may or may not be part of one's body, decreasing the incorporation of the rubber hand while it increased the incorporation of the neutral object in the RHI paradigm (Tsakiris, Costantini, & Haggard, 2008).

Taken together, our results are also coherent with findings on brain‐damaged patients. Neuropsychological investigations are very relevant in supporting the idea that the regions found in the current study contribute to the emergence of a bodily self‐awareness. For instance, lesions to the right‐lateralized set of areas here identified could induce Unilateral Spatial Neglect (USN), a syndrome characterized by the inability to attend to stimuli from the contralesional side of space frequently associated with complex body representation disorders, such as somatoparaphrenia (Gandola et al., 2012; Invernizzi et al., 2013; Vallar & Ronchi, 2009). Damage to the inferior parietal lobule, in particular, is more frequently associated with the personal, bodily components of USN, such as personal neglect (Committeri et al., 2007; Vallar & Calzolari, 2018).

Furthermore, it has been recently suggested that the subjective experience of an external event (e.g., spatial perception) would result from the neural responses to visceral activity such as heartbeats. The integration of visceral input with visual perception would provide visual content with a first‐person perspective (Tallon‐Baudry, Campana, Park, & Babo‐Rebelo, 2018). Tallon‐Baudry et al. (2018) identified a brain network that may be responsible for the interplay between neural signals generated by heartbeats and visual awareness: this includes the rTPJ, the same area found by us. Its damage or disconnection can lead to USN (see Chelazzi, Bisley, & Bartolomeo, 2018) but also to the so‐called “out‐of‐body experience,” a situation in which patients perceive themselves as observing their body from an extracorporeal perspective (Blanke, Landis, Spinelli, & Seeck, 2004). Also, lesions or structural alterations to insular regions have been associated with both body ownership and interoceptive disorders (ereda, Ghika, Maeder, & Bogousslavsky, 2002; Ibañez et al., 2010; Karnath, 2005; Moro et al., 2016; Ronchi et al., 2015; Salvato, Mercurio, Sberna, Paulesu, & Bottini, 2018).

Interestingly, left caloric vestibular stimulation (CVS), associated with insular activations, may induce a temporary remission of both symptoms, also modulating body temperature, as it was previously found in a single right brain‐damaged patient suffering from chronic somatoparaphrenia: the restored sense of limb ownership following CVS was associated with an increase of the body temperature (Salvato, Gandola, et al., 2018). Notably, left CVS acts on the brain areas found here (Lopez et al., 2012; Zu Eulenburg, Caspers, Roski, & Eickhoff 2012), a fact that fits well with a role of these areas in creating a point of contact between a sense of body ownership and interoception.

Finally, it is also important to note that our results partially confirmed the hypotheses suggested in two theoretical papers on the topic (Moseley et al., 2012; Park & Blanke, 2019), in which an integrated system processing exteroceptive and interoceptive signals has been proposed. Such system would involve the parietal cortex and the insula (Moseley et al., 2012), or two subnetworks involving the premotor cortex, TPJ, intraparietal sulcus, posterior cingulate cortex, and the insula, overlapping in the intraparietal sulcus (Park & Blanke, 2019). If on the one hand, we confirmed the role of the parietal regions, precentral and postcentral gyri, and TPJ, on the other hand, we did not find any direct cluster of convergence in the insular cortex. Yet, our SMGs clusters also expand to the Rolandic operculi and posterior insular cortices. Functional activity of the posterior insula and nearby regions is associated with changes in somatosensory processing linked to altered states of bodily self‐awareness (e.g., illusory self‐identification with an avatar; Allison, Wood, McCarthy, & Spencer, 1991; Bufalari, Aprile, Avenanti, Di Russo, & Aglioti, 2007). These regions are strongly multimodal: activity within the posterior insula and parietal operculum were found to be increased by several bodily related stimuli such as touch, noxious heat, and CVS (Zu Eulenburg, Baumgärtner, Treede, & Dieterich, 2013).

## 5 CONCLUSIONS AND FUTURE DIRECTIONS

In conclusion, our data indicated that external and internal signals might converge in the SMG bilaterally together with a right‐lateralized set of areas such as the precentral, postcentral, and superior temporal gyri. These higher‐order brain areas are involved in integrating multisensory signals, and in recalibrating information from different incoming channels and spatial frames of reference.

Although disorders of body ownership are on record since 1942 (Gerstmann, 1942), and disorders of interoception have been repeatedly described (Critchley & Garfinkel, 2018; Salvato, Mercurio, et al., 2018), it remains to be investigated whether a higher order deficit of the bodily self‐awareness exists with specific deficits due to a perturbed integration of the two dimensions. Our results set the rationale for future neuropsychological and brain stimulation studies that may explore the contribution and weight of each area found in this meta‐analysis in the integration of the bodily self‐awareness.

## CONFLICT OF INTEREST

The authors declare no conflict of interest.

## Supporting information


**Appendix S1:** Supporting information

## Data Availability

The data that support the findings of this study are openly available (see Supporting Information).

## Endnote

1 The regional quantification of the spatial extent of the clusters was performed by comparison with AAL atlas (Tzourio‐Mazoyer et al., 2002).
