# Supplementary material for: Building the bodily self‐awareness: Evidence for the convergence between interoceptive and exteroceptive information in a multilevel kernel density analysis study
Source: Hum Brain Mapp. 2019 Oct 14;41(2):401–18. doi: 10.1002/hbm.24810 (PMC7268061; doi:10.1002/hbm.24810)
Supplement: Supplementary file 1 — Appendix S1: Supporting information [file HBM-41-401-s001.docx]

*Supplementary materials for:*

“**Building the sense of the self: the convergence between interoceptive and exteroceptive information in a Multilevel Kernel Density Analysis study** "

Gerardo, Salvato^1,2,3^*; Fabian, Richter^4^*; Lucas, Sedeño^5,6^; Gabriella, Bottini^1,2,3^; Eraldo, Paulesu^7,8^

^1^ Department of Brain and Behavioural Sciences, University of Pavia, Pavia, Italy

^2^ Centre of Cognitive Neuropsychology, ASST Grande Ospedale Metropolitano, Niguarda Hospital, Milano, Italy

^3^ NeuroMI, Milan Center for Neuroscience, Milan, Italy

^4^ Universität zu Köln, Department of Psychology, Cologne, Germany

^5^ Laboratory of Experimental Psychology and Neuroscience (LPEN), Institute of Translational and Cognitive Neuroscience (INCyT), INECO Foundation, Favaloro University, Buenos Aires, Argentina

^6^ National Scientific and Technical Research Council (CONICET), Buenos Aires, Argentina

^7^ Department of Psychology, University of Milano-Bicocca, Milano, Italy

^8^ IRCCS Istituto Ortopedico Galeazzi, Milano, Italy

*The MKDA approach*

The MKDA emphasises the multi-level hierarchy of the meta-analytic input data by treating activation peaks as nested within contrasts. The resulting contrast maps instead of individual peaks are the unit of analysis, and through this, two levels of analysis (within-contrasts and between contrasts) are created. So, the MKDA summarises consistency across studies rather than just summarising consistencies across peak coordinates. Analogous to individual functional imaging studies and following the terminology of Borenstein and colleagues (Borenstein, Hedges, Higgins, & Rothstein, 2010), the MKDA is hereby a ‘random effects’ approach. In random-effects models, subjects - instead of observations (fixed-effects models) - constitute the unit of analysis [Fan, Duncan, de Greck, & Northoff, 2011; Kober & Wager, 2010; Wager et al., 2009). An important consequence is that any single study that reports a large number of nearby peaks (due to differences in reporting, voxel size, thresholding or low spatial smoothness in the data) cannot dramatically influence the analyses (Wager et al., 2008).

The meta-analyses were performed in MatlabR2012a (Mathworks, Naticks, MA) using the MKDA tool package created by (Wager et al., 2009) (http://wagerlab.colorado.edu/files/tools/meta-analysis.html). First, comparison indicator maps (CIMs) were created for each set of coordinates by convolving a 10-mm spherical kernel around each peak reported in this contrast and plotting these kernel-regions onto a canonical brain (avg152T1.img; SPM, Wellcome Department of Imaging Neuroscience). Note that, before the analysis, all peak coordinates were converted into a common stereotactic space (Montreal Neurological Institute (MNI)), which is widely used in the field and minimises localisation errors in meta-analytical results (Carmack et al., 2004). The resulting binary CIMs (“active” voxels were given a value of 1) contained voxels that were within 10 mm of a reported peak. This procedure also reduces potential bias introduced by studies with many close-by peaks, because the neighbouring peaks will contribute to just one overlapping region with a maximum activity of 1.

Spherical kernels of 10 mm were chosen because they match roughly the three dimensional spatial resolution of the reported data and due to its generally acceptance in MKDA use (Kober & Wager, 2010; Salimi-Khorshidi, Smith, Keltner, Wager, & Nichols, 2009; Wager, Jonides, & Reading, 2004; Wager et al., 2007). Coordinates originally reported in Talairach stereotactic space were converted to MNI space, which is widely used in the field and minimises localisation errors in meta-analytical results (Carmack et al., 2004).

In the next step, a density map reflecting the proportion of contrasts that activate near each voxel (MKDA uses a spatial resolution of 2 x 2 x 2 mm per voxel) was created by taking a weighted average (weights were sample size and type of effect) of contrast activation maps. This density map was then assessed for statistical significance against a null hypothesis stating that activated regions are randomly distributed across the grey matter of the standard brain. To identify voxels with activations that exceed the frequency expected by chance, a threshold derived from a Monte Carlo Simulation with 5000 iterations per analysis was used. During these simulations, the observed number of activations from the CIMs were placed at random locations throughout the brain. Building on this, MKDA identifies maps of activated clusters according to a ‘height’ or ‘extent’ based threshold. The height-based threshold encloses voxels that have proportions of contrasts inside the 10 mm kernel region that exceed the maximum expected over the entire brain by chance (*p* < .05, family Wise Error rate - FWE corrected) and reducing to this level the chance of making a Type I error at any single voxel to less than or equal to 5 per cent.

Additionally, to determine a cluster extent-based threshold, the largest cluster of contiguous voxels was saved after each Monte Carlo iteration. So, the extent-based threshold selects contiguous voxels (cluster) outside the 10 mm sphere of the peaks found using the height-based threshold, that show greater activation than would be expected at a given level of chance (*p* < .05) and which are secondary FWE-corrected for spatial extent at *p* < .05. Beyond that, a combined map of voxels meeting both criteria (height and extent) was computed using SPM8 contiguity assessment procedures.

In sum, the Contrast Indicator Maps (CIMs), in which voxels are given a value of 1 if they were “active” and 0 if not, are combined to form a single nested map for each contrast. This guarantees that each contrast contributes no more than a value of 1 for a given voxel when overall proportion statistics are calculated. These combined CIMs are then weighted by the square root of the sample size of the contrast, reflecting the proportion of contrasts yielding activations near each voxel. The weighting factors are computed by multiplying the square root of *N* (sample size) with 0.75 for fixed-effects versus 1.00 for random-effects analysis. This adjustment is reflected in the following formula:

*Equation 1*

$$P = \sum_{c}^{\infty} Ic\left( \frac{\delta c\sqrt{Nc}}{\begin{aligned} \sum_{c}^{\infty} \delta c\sqrt{Nc} \\ \end{aligned}} \right)$$

*P* is the weighted proportion of CIMs that activated within 10 mm of a certain voxel, *c* indexes comparison maps *I*, *δ* is the fixed effects discounting factor, and *N* is the sample size.

CIMs from one condition can then be compared with those from another condition. The meta-analysis statistic at each voxel is the proportion difference of contrasts that activated within 10 mm of that voxel, weighted by the sample size of the study (Buhle et al., 2014; Denny, Kober, Wager, & Kevin N. Ochsner, 2012; Kober et al., 2008; Mende-Siedlecki et al., 2013).

The resulting coordinates responding to brain areas showing significant activation were localized with: Multi-image Analysis GUI (Mango version 2.6, University of Texas Health Science Center, San Antonio; http://ric.uthscsa.edu/mango/) by overlaying significant voxels on the MNI average template; the AAL atlas (Tzourio-Mazoyer et al., 2002) using MRIcron; and Brede Database (Technical University of Denmark, Copenhagen; <http://neuro.imm.dtu.dk/services/brededatabase/>). Using these different atlases and methods produced more accurate results than automated labelling systems. Also, we chose to use the MNI template brain as it is a common standard template for electronic registration, and thus using it minimises localisation errors in the meta-analysis results.

| Area | x | y | z | Brodmann Area | Voxels | Volume (mm^3^) | Maxstat (z) |
| --- | --- | --- | --- | --- | --- | --- | --- |
| *Height Threshold (p < .05, family wise error rate - FWE corrected)* | | | | | | | |
| Right Insula | 40 | 8 | 4 | 13 | 528 | 4224 | 0.23 |
|  | 42 | 2 | 2 |  | 118 |  | 0.2 |
|  | 42 | 10 | 2 |  | 350 |  | 0.23 |
|  | 38 | -4 | 12 |  | 8 |  | 0.14 |
|  | 36 | 6 | 12 |  | 52 |  | 0.19 |
| Left Insula | -38 | 8 | -6 | 13 | 2 | 16 | 0.14 |
| Mid Cingulate Gyrus | 2 | 8 | 42 | 24 | 1 | 8 | 0.14 |
| Mid Cingulate Gyrus | 0 | 6 | 38 | 24 | 1 | 8 | 0.14 |
| Left Insula | -40 | 4 | 4 | 13 | 143 | 1144 | 0.19 |
| Mid Cingulate Gyrus | 2 | 2 | 48 | 24 | 104 | 832 | 0.15 |
| Right Insula | 44 | -8 | 10 | 13 | 4 | 32 | 0.15 |
| Right Claustrum | 42 | -14 | 4 |  | 2 | 16 | 0.15 |
| Right Thalamus | 16 | -16 | 16 |  | 16 | 128 | 0.13 |
| Right Insula | 42 | -18 | 4 | 13 | 1 | 8 | 0.13 |
| *Extent Threshold (primary alpha level of p < .001)* | | | | | | | |
| Anterior Cingulate | 0 | 30 | 18 | 24 | 251 | 2008 | 0.12 |
| Left Globus Pallidus (Lentiform Ncl.) | -22 | -14 | -12 |  | 169 | 1352 | 0.13 |
| Right Postcentral Gyrus | 20 | -28 | 60 | 4 | 187 | 1496 | 0.10 |

**Table S3. Interoception > Body Ownership meta-analysis results**. Stereotactic coordinates for the most consistent peak activation foci for meta-analytic Interoception > Body Ownership subtraction analysis. Peak is the location of peak activation in the cluster; coordinates are reported in MNI space; Maxstat (z) is the weighted percentage of contrasts that activated in each cluster.

| Area | x | y | z | Brodmann Area | Voxels | Volume (mm^3^) | Maxstat (z) |
| --- | --- | --- | --- | --- | --- | --- | --- |
| *Height Threshold (p < .05, family-wise error rate - FWE corrected)* | | | | | | | |
| Right Mid Occipital Gyrus | 52 | -56 | -6 | 19 | 23 | 184 | 0.17 |
| Left Inferior Temporal Gyrus (Occipital Lobe) | -46 | -68 | -2 | 37 | 12 | 96 | 0.15 |
| Left Occipital Inferior Lobe (Fusiform Gyrus) | -40 | -72 | -10 | 19 | 18 | 144 | 0.17 |
| *Extent Threshold (primary alpha level of p < .001)* | | | | | | | |
| Left Precentral Gyrus | -50 | 0 | 36 | 6 | 281 | 2248 | 0.12 |
| Right Inferior Parietal Lobe | 56 | -30 | 48 | 40 | 185 | 1480 | 0.13 |
| Right Cerebellum (Tonsil) | 30 | -68 | -34 |  | 214 | 1712 | 0.12 |

**Table S4. Body Ownership > Interoception meta-analysis results.** Stereotactic coordinates for the most consistent peak activation foci for meta-analytic Body Ownership > Interoception subtraction analysis. Peak is the location of peak activation in the cluster; coordinates are reported in MNI space; Maxstat (z) is the weighted percentage of contrasts that activated in each cluster.

**References:**

Araujo, H. F., Kaplan, J., Damasio, H., & Damasio, A. (2015). Neural correlates of different self domains. *Brain and Behavior*, *5*(12), 1–5. https://doi.org/10.1002/brb3.409

Atlas, L. Y., & Wager, T. D. (2014). A meta-analysis of brain mechanisms of placebo analgesia: Consistent findings and unanswered questions. *Handbook of Experimental Pharmacology*, *225*, 37–69. https://doi.org/10.1007/978-3-662-44519-8_3

Avery, J. A., Kerr, K. L., Ingeholm, J. E., Burrows, K., Bodurka, J., & Simmons, W. K. (2015). A common gustatory and interoceptive representation in the human mid-insula. *Human Brain Mapping*, *36*(8), 2996–3006. https://doi.org/10.1002/hbm.22823

Bauer, C. C. C., Díaz, J. L., Concha, L., & Barrios, F. A. (2014). Sustained attention to spontaneous thumb sensations activates brain somatosensory and other proprioceptive areas. *Brain and Cognition*, *87*(1), 86–96. https://doi.org/10.1016/j.bandc.2014.03.009

Becker, C. A., Schmälzle, R., Flaisch, T., Renner, B., & Schupp, H. T. (2014). Thirst and the state-dependent representation of incentive stimulus value in human motive circuitry. *Social Cognitive and Affective Neuroscience*, *10*(12), 1722–1729. https://doi.org/10.1093/scan/nsv063

Bekrater-Bodmann, R., Foell, J., Diers, M., Kamping, S., Rance, M., Kirsch, P., … Flor, H. (2014). The importance of synchrony and temporal order of visual and tactile input for illusory limb ownership experiences - An fMRI study applying virtual reality. *PLoS ONE*, *9*(1). http://doi.org/10.1371/journal.pone.0087013

Binks, A. P., Evans, K. C., Reed, J. D., Moosavi, S. H., & Banzett, R. B. (2014). The time-course of cortico-limbic neural responses to air hunger. *Respiratory Physiology and Neurobiology*, *204*, 78–85. https://doi.org/10.1016/j.resp.2014.09.005

Borenstein, M., Hedges, L. V., Higgins, J. P. T., & Rothstein, H. R. (2010). A basic introduction to fixed-effect and random-effects models for meta-analysis. *Research Synthesis Methods*, *1*(2), 97–111. https://doi.org/10.1002/jrsm.12

Brannan, S., Liotti, M., Egan, G., Shade, R., Madden, L., Robillard, R., … Fox, P. T. (2001). Neuroimaging of cerebral activations and deactivations associated with hypercapnia and hunger for air. *Proceedings of the National Academy of Sciences*, *98*(4), 2029–2034. https://doi.org/10.1073/pnas.98.4.2029

Brozzoli, C., Gentile, G., & Ehrsson, H. H. (2012). That’s Near My Hand! Parietal and Premotor Coding of Hand-Centered Space Contributes to Localization and Self-Attribution of the Hand. *Journal of Neuroscience*, *32*(42), 14573–14582. http://doi.org/10.1523/JNEUROSCI.2660-12.2012

Buhle, J. T., Silvers, J. A., Wage, T. D., Lopez, R., Onyemekwu, C., Kober, H., … Ochsner, K. N. (2014). Cognitive reappraisal of emotion: A meta-analysis of human neuroimaging studies. *Cerebral Cortex*, *24*(11), 2981–2990. https://doi.org/10.1093/cercor/bht154

Cacioppo, S., Frum, C., Asp, E., Weiss, R. M., Lewis, J. W., & Cacioppo, J. T. (2013). A quantitative meta-analysis of functional imaging studies of social rejection. *Scientific Reports*, *3*. https://doi.org/10.1038/srep02027

Cameron, O. G., & Minoshima, S. (2002). Regional brain activation due to pharmacologically induced adrenergic interoceptive stimulation in humans. *Psychosomatic Medicine*, *64*(6), 851–861. https://doi.org/10.1097/01.PSY.0000038939.33335.32

Carmack, P. S., Spence, J., Gunst, R. F., Schucany, W. R., Woodward, W. A., & Haley, R. W. (2004). Improved agreement between Talairach and MNI coordinate spaces in deep brain regions. *NeuroImage*, *22*(1), 367–371. https://doi.org/10.1016/j.neuroimage.2004.01.022

Caseras, X., Murphy, K., Mataix-Cols, D., López-Solà, M., Soriano-Mas, C., Ortriz, H., … Torrubia, R. (2013). Anatomical and functional overlap within the insula and anterior cingulate cortex during interoception and phobic symptom provocation. *Human Brain Mapping*, *34*(5), 1220–1229. https://doi.org/10.1002/hbm.21503

Coen, S. J., Yágüez, L., Aziz, Q., Mitterschiffthaler, M. T., Brammer, M., Williams, S. C. R., & Gregory, L. J. (2009). Negative Mood Affects Brain Processing of Visceral Sensation. *Gastroenterology*, *137*(1). https://doi.org/10.1053/j.gastro.2009.02.052

Critchley, H. D., Wiens, S., Rotshtein, P., Öhman, A., & Dolan, R. J. (2004). Neural systems supporting interoceptive awareness. *Nature Neuroscience*, *7*(2), 189–195. https://doi.org/10.1038/nn1176

Denny, B. T., Kober, H., Wager, T. D., & Kevin N. Ochsner. (2012). A meta-analysis of functional neuroimaging studies of self-and other judgments reveals a spatial gradient for mentalizing in medial prefrontal cortex. *Journal of Cognitive Neuroscience*, *24*(8), 1742–1752. https://doi.org/10.1162/jocn_a_00233

Denton, D. A., Shade, R., Zamarippa, F., Egan, G., Blair-West, J., McKinley, M. J., & Fox, P. (1999). Correlation of regional cerebral blood flow and change of plasma sodium concentration during genesis and satiation of thirst. *Proceedings of the National Academy of Sciences of the United States of America*, *96*(5), 2532–2537. https://doi.org/10.1073/pnas.96.5.2532

Denton, D., Shade, R., Zamarippa, F., Egan, G., Blair-West, J., McKinley, M., … Fox, P. (1999). Neuroimaging of genesis and satiation of thirst and an interoceptor-driven theory of origins of primary consciousness. *Proceedings of the National Academy of Sciences*, *96*(9), 5304–5309. https://doi.org/10.1073/pnas.96.9.5304

Egan, G., Silk, T., Zamarripa, F., Williams, J., Federico, P., Cunnington, R., … Denton, D. (2003). Neural correlates of the emergence of consciousness of thirst. *Proceedings of the National Academy of Sciences*, *100*(25), 15241–15246. https://doi.org/10.1073/pnas.2136650100

Ehrsson, H. H., Holmes, N. P., & Passingham, R. E. (2005). Touching a rubber hand: feeling of body ownership is associated with activity in multisensory brain areas. *The Journal of Neuroscience : The Official Journal of the Society for Neuroscience*, *25*(45), 10564–73. http://doi.org/10.1523/JNEUROSCI.0800-05.2005

Ehrsson, H. H., Spence, C., & Passingham, R. E. (2004). That’s my hand! Activity in premotor cortex reflects feeling of ownership of a limb. *Science (New York, N.Y.)*, *305*(5685), 875–7. http://doi.org/10.1126/science.1097011

Ernst, J., Northoff, G., Böker, H., Seifritz, E., & Grimm, S. (2013). Interoceptive awareness enhances neural activity during empathy. *Human Brain Mapping*, *34*(7), 1615–1624. https://doi.org/10.1002/hbm.22014

Evans, K. C., Banzett, R. B., Adams, L., McKay, L., Frackowiak, R. S. J., & Corfield, D. R. (2002). BOLD fMRI identifies limbic, paralimbic, and cerebellar activation during air hunger. *Journal of Neurophysiology*, *88*(3), 1500–1511. https://doi.org/10.1152/jn.00957.2001

Fan, Y., Duncan, N. W., de Greck, M., & Northoff, G. (2011). Is there a core neural network in empathy? An fMRI based quantitative meta-analysis. *Neuroscience and Biobehavioral Reviews*. https://doi.org/10.1016/j.neubiorev.2010.10.009

Farb, N. A. S., Segal, Z. V., & Anderson, A. K. (2013). Mindfulness meditation training alters cortical representations of interoceptive attention. *Social Cognitive and Affective Neuroscience*, *8*(1), 15–26. https://doi.org/10.1093/scan/nss066

Farrell, M. J., Egan, G. F., Zamarripa, F., Shade, R., Blair-West, J., Fox, P., & Denton, D. A. (2006). Unique, common, and interacting cortical correlates of thirst and pain. *Proceedings of the National Academy of Sciences*, *103*(7), 2416–2421. https://doi.org/10.1073/pnas.0511019103

Gentile, G., Björnsdotter, M., Petkova, V. I., Abdulkarim, Z., & Ehrsson, H. H. (2015). Patterns of neural activity in the human ventral premotor cortex reflect a whole-body multisensory percept. *NeuroImage*, *109*, 328–340. http://doi.org/10.1016/j.neuroimage.2015.01.008

Gentile, G., Guterstam, A., Brozzoli, C., & Ehrsson, H. H. (2013). Disintegration of Multisensory Signals from the Real Hand Reduces Default Limb Self-Attribution: An fMRI Study. *Journal of Neuroscience*, *33*(33), 13350–13366. http://doi.org/10.1523/JNEUROSCI.1363-13.2013

Guterstam, A., Björnsdotter, M., Gentile, G., & Ehrsson, H. H. (2015). Posterior cingulate cortex integrates the senses of self-location and body ownership. *Current Biology*, *25*(11), 1416–1425. http://doi.org/10.1016/j.cub.2015.03.059

Guterstam, A., Gentile, G., & Ehrsson, H. H. (2013). The Invisible Hand Illusion: Multisensory Integration Leads to the Embodiment of a Discrete Volume of Empty Space. *Journal of Cognitive Neuroscience*, *25*(7), 1078–1099. http://doi.org/10.1162/jocn_a_00393

Haase, L., May, A. C., Falahpour, M., Isakovic, S., Simmons, A. N., Hickman, S. D., … Paulus, M. P. (2015). A pilot study investigating changes in neural processing after mindfulness training in elite athletes. *Frontiers in Behavioral Neuroscience*, *9*. https://doi.org/10.3389/fnbeh.2015.00229

Haase, L., Stewart, J. L., Youssef, B., May, A. C., Isakovic, S., Simmons, A. N., … Paulus, M. P. (2016). When the brain does not adequately feel the body: Links between low resilience and interoception. *Biological Psychology*, *113*, 37–45. https://doi.org/10.1016/j.biopsycho.2015.11.004

Immordino-Yang, M. H., Yang, X.-F., & Damasio, H. (2014). Correlations between social-emotional feelings and anterior insula activity are independent from visceral states but influenced by culture. *Frontiers in Human Neuroscience*, *8*. https://doi.org/10.3389/fnhum.2014.00728

Ionta, S., Heydrich, L., Lenggenhager, B., Mouthon, M., Fornari, E., Chapuis, D., … Blanke, O. (2011). Multisensory Mechanisms in Temporo-Parietal Cortex Support Self-Location and First-Person Perspective. *Neuron*, *70*(2), 363–374. http://doi.org/10.1016/j.neuron.2011.03.009

Isaev, G., Murphy, K., Guz, A., & Adams, L. (2002). Areas of the brain concerned with ventilatory load compensation in awake man. *Journal of Physiology*, *539*(3), 935–945. https://doi.org/10.1113/jphysiol.2001.012957

Kober, H., & Wager, T. D. (2010). Meta-analysis of neuroimaging data. *Wiley Interdisciplinary Reviews: Cognitive Science*, *1*(2), 293–300. https://doi.org/10.1002/wcs.41

Kober, H., Barrett, L. F., Joseph, J., Bliss-Moreau, E., Lindquist, K., & Wager, T. D. (2008). Functional grouping and cortical-subcortical interactions in emotion: A meta-analysis of neuroimaging studies. *NeuroImage*, *42*(2), 998–1031. https://doi.org/10.1016/j.neuroimage.2008.03.059

Kuehn, E., Mueller, K., Lohmann, G., & Schuetz-Bosbach, S. (2016). Interoceptive awareness changes the posterior insula functional connectivity profile. *Brain Structure and Function*, *221*(3), 1555–1571. https://doi.org/10.1007/s00429-015-0989-8

Limanowski, J., & Blankenburg, F. (2015). Network activity underlying the illusory self-attribution of a dummy arm. *Human Brain Mapping*, *36*(6), 2284–2304. http://doi.org/10.1002/hbm.22770

Limanowski, J., & Blankenburg, F. (2016). That’s not quite me: Limb ownership encoding in the brain. *Social Cognitive and Affective Neuroscience*, *11*(7), 1130–1140. <http://doi.org/10.1093/scan/nsv079>

Limanowski, J., & Blankenburg, F. (2018). Fronto-parietal brain responses to visuotactile congruence in an anatomical reference frame. Frontiers in Human Neuroscience 12, 48.

Lindquist, K. A., Satpute, A. B., Wager, T. D., Weber, J., & Barrett, L. F. (2016). The Brain Basis of Positive and Negative Affect: Evidence from a Meta-Analysis of the Human Neuroimaging Literature. *Cerebral Cortex*, *26*(5), 1910–1922. https://doi.org/10.1093/cercor/bhv001

Liotti, M., Brannan, S., Egan, G., Shade, R., Madden, L., Abplanalp, B., … Denton, D. (2001). Brain responses associated with consciousness of breathlessness (air hunger). *Proceedings of the National Academy of Sciences*, *98*(4), 2035–2040. https://doi.org/10.1073/pnas.98.4.2035

May, A. C., Stewart, J. L., Migliorini, R., Tapert, S. F., & Paulus, M. P. (2013). Methamphetamine Dependent Individuals Show Attenuated Brain Response to Pleasant Interoceptive Stimuli. *Drug and Alcohol Dependence*, *131*(3), 238–246. https://doi.org/10.1016/j.drugalcdep.2013.05.029

May, A. C., Stewart, J. L., Tapert, S. F., & Paulus, M. P. (2014). The effect of age on neural processing of pleasant soft touch stimuli. *Frontiers in Behavioral Neuroscience*, *8*. https://doi.org/10.3389/fnbeh.2014.00052

Mende-Siedlecki, P., Said, C. P., & Todorov, A. (2013). The social evaluation of faces: a meta-analysis of functional neuroimaging studies. *Soc Cogn Affect Neurosci*, *8*(3), 285–299. https://doi.org/10.1093/scan/nsr090

Migliorini, R., Stewart, J. L., May, A. C., Tapert, S. F., & Paulus, M. P. (2013). What do you feel? Adolescent drug and alcohol users show altered brain response to pleasant interoceptive stimuli. *Drug and Alcohol Dependence*, *133*(2), 661–668. https://doi.org/10.1016/j.drugalcdep.2013.08.015

Oberndorfer, T., Simmons, A., McCurdy, D., Strigo, I., Matthews, S., Yang, T., … Kaye, W. (2013). Greater anterior insula activation during anticipation of food images in women recovered from anorexia nervosa versus controls. *Psychiatry Research - Neuroimaging*, *214*(2), 132–141. https://doi.org/10.1016/j.pscychresns.2013.06.010

Perini, I., Morrison, I., & Olausson, H. (2015). Seeking pleasant touch: neural correlates of behavioral preferences for skin stroking. *Frontiers in Behavioral Neuroscience*, *9*. https://doi.org/10.3389/fnbeh.2015.00008

Petkova, V. I., Björnsdotter, M., Gentile, G., Jonsson, T., Li, T. Q., & Ehrsson, H. H. (2011). From part- to whole-body ownership in the multisensory brain. *Current Biology*, *21*(13), 1118–1122. http://doi.org/10.1016/j.cub.2011.05.022

Pollatos, O., Schandry, R., Auer, D. P., & Kaufmann, C. (2007). Brain structures mediating cardiovascular arousal and interoceptive awareness. *Brain Research*, *1141*(1), 178–187. https://doi.org/10.1016/j.brainres.2007.01.026

Preston, C., & Ehrsson, H. H. (2016). Illusory obesity triggers body dissatisfaction responses in the insula and anterior cingulate cortex. *Cerebral Cortex*, *26*(12), 4450–4460. http://doi.org/10.1093/cercor/bhw313

Salimi-Khorshidi, G., Smith, S. M., Keltner, J. R., Wager, T. D., & Nichols, T. E. (2009). Meta-analysis of neuroimaging data: A comparison of image-based and coordinate-based pooling of studies. *NeuroImage*, *45*(3), 810–823. https://doi.org/10.1016/j.neuroimage.2008.12.039

Schurz, M., Radua, J., Aichhorn, M., Richlan, F., & Perner, J. (2014). Fractionating theory of mind: A meta-analysis of functional brain imaging studies. *Neuroscience and Biobehavioral Reviews*. https://doi.org/10.1016/j.neubiorev.2014.01.009

Simmons, W. K., Avery, J. A., Barcalow, J. C., Bodurka, J., Drevets, W. C., & Bellgowan, P. (2013). Keeping the body in mind: Insula functional organization and functional connectivity integrate interoceptive, exteroceptive, and emotional awareness. *Human Brain Mapping*, *34*(11), 2944–2958. https://doi.org/10.1002/hbm.22113

Stewart, J. L., Juavinett, A. L., May, A. C., Davenport, P. W., & Paulus, M. P. (2015). Do you feel alright? Attenuated neural processing of aversive interoceptive stimuli in current stimulant users. *Psychophysiology*, *52*(2), 249–262. https://doi.org/10.1111/psyp.12303

Stewart, J. L., May, A. C., Poppa, T., Davenport, P. W., Tapert, S. F., & Paulus, M. P. (2014). You are the danger: Attenuated insula response in methamphetamine users during aversive interoceptive decision-making. *Drug and Alcohol Dependence*, *142*, 110–119. https://doi.org/10.1016/j.drugalcdep.2014.06.003

Stewart, J. L., Parnass, J. M., May, A. C., Davenport, P. W., & Paulus, M. P. (2013). Altered frontocingulate activation during aversive interoceptive processing in young adults transitioning to problem stimulant use. *Frontiers in Systems Neuroscience*, *7*. https://doi.org/10.3389/fnsys.2013.00089

Strigo, I. A., Matthews, S. C., Simmons, A. N., Oberndorfer, T., Klabunde, M., Reinhardt, L. E., & Kaye, W. H. (2013). Altered insula activation during pain anticipation in individuals recovered from anorexia nervosa: Evidence of interoceptive dysregulation. *International Journal of Eating Disorders*, *46*(1), 23–33. https://doi.org/10.1002/eat.22045

Terasawa, Y., Fukushima, H., & Umeda, S. (2013). How does interoceptive awareness interact with the subjective experience of emotion? An fMRI Study. *Human Brain Mapping*, *34*(3), 598–612. https://doi.org/10.1002/hbm.21458

Tracy, J., Goyal, N., Flanders, A., Weening, R., Laskas, J., Natale, P., & Waldron, B. (2007). Functional magnetic resonance imaging analysis of attention to one’s heartbeat. In *Psychosomatic Medicine* (Vol. 69, pp. 952–960). https://doi.org/10.1097/PSY.0b013e31815b60cf

Tsakiris, M., Hesse, M. D., Boy, C., Haggard, P., & Fink, G. R. (2007). Neural signatures of body ownership: A sensory network for bodily self-consciousness. *Cerebral Cortex*, *17*(10), 2235–2244. http://doi.org/10.1093/cercor/bhl131

Tsakiris, M., Longo, M. R., & Haggard, P. (2010). Having a body versus moving your body: Neural signatures of agency and body-ownership. *Neuropsychologia*, *48*(9), 2740–2749. http://doi.org/10.1016/j.neuropsychologia.2010.05.021

Tzourio-Mazoyer, N., Landeau, B., Papathanassiou, D., Crivello, F., Etard, O., Delcroix, N., … Joliot, M. (2002). Automated anatomical labeling of activations in SPM using a macroscopic anatomical parcellation of the MNI MRI single-subject brain. *NeuroImage*, *15*(1), 273–289. https://doi.org/10.1006/nimg.2001.0978

Wager, T. D., Barrett, L. F., Bliss-moreau, E., Lindquist, K. A., Duncan, S., Kober, H., … Mize, J. (2008). The Neuroimaging of Emotion. *Handbook of Emotions*, (212), 249–271. https://doi.org/10.2307/2076468

Wager, T. D., Jonides, J., & Reading, S. (2004). Neuroimaging studies of shifting attention: A meta-analysis. *NeuroImage*, *22*(4), 1679–1693. https://doi.org/10.1016/j.neuroimage.2004.03.052

Wager, T. D., Lindquist, M. A., Nichols, T. E., Kober, H., & Van Snellenberg, J. X. (2009). Evaluating the consistency and specificity of neuroimaging data using meta-analysis. *NeuroImage*. https://doi.org/10.1016/j.neuroimage.2008.10.061

Wager, T. D., Lindquist, M., & Kaplan, L. (2007). Meta-analysis of functional neuroimaging data: Current and future directions. *Social Cognitive and Affective Neuroscience*, *2*(2), 150–158. https://doi.org/10.1093/scan/nsm015

Wang, G. J., Tomasi, D., Backus, W., Wang, R., Telang, F., Geliebter, A., … Volkow, N. D. (2008). Gastric distention activates satiety circuitry in the human brain. *NeuroImage*, *39*(4), 1824–1831. https://doi.org/10.1016/j.neuroimage.2007.11.008

Zaki, J., Davis, J. I., & Ochsner, K. N. (2012). Overlapping activity in anterior insula during interoception and emotional experience. *NeuroImage*, *62*(1), 493–499. https://doi.org/10.1016/j.neuroimage.2012.05.012
